# Deep Eutectic Solvent Stir Bar Sorptive Extraction: A Rapid Microextraction Technique for the Determination of Vitamin D_3_ by Spectrophotometry

**DOI:** 10.1021/acsomega.3c01670

**Published:** 2023-06-23

**Authors:** Sayyed Hossein Hashemi, Massoud Kaykhaii, Ahmad Jamali Keikha, Leila Raisi

**Affiliations:** † Department of Marine Chemistry, Faculty of Marine Science, Chabahar Maritime University, Chabahar, Iran; ‡ Department of Process Engineering and Chemical Technology, Faculty of Chemistry, Gdańsk University of Technology, Narutowicza 11/12 St., Gdansk 80-233, Poland; § Department of Mechanical Engineering, Faculty of Marine Engineering, Chabahar Maritime University, Chabahar, Iran

## Abstract

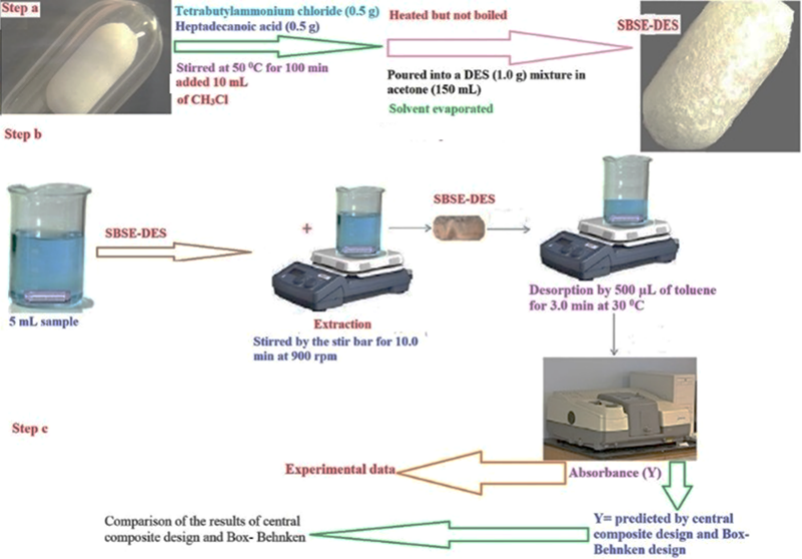

In this paper, a new microextraction method, named deep eutectic solvent stir bar sorptive extraction (DES–SBSE), is described that utilizes a hydrophobic DES (hDES) as a coating for SBSE. As a model, vitamin D_3_ was extracted efficiently by this technique from different real samples before its spectrophotometric determination. A conventional magnet was encapsulated inside a glass bar (1.0 cm × 2 mm) and coated by the hDES consisting of tetrabutylammonium chloride and heptadecanoic acid (mole ratio 1:2). Parameters affecting microextraction were studied and optimized by one-variable-at-a-time, central composite design, and Box–Behnken design methods. Under the optimum conditions, the detection limit of 0.08 μg L^–1^ could be reached. The linear range of the method was between 0.5 and 1000.0 μg L^–1^ for the analyte. Precision for intraday repeatability and interday reproducibility of the method was better than 3.1 and 4.2, respectively. A single stir bar could be used for at least 50 successive extractions and the batch-to-batch reproducibility of SB coated with hDES was 4.5%.

## 1 Introduction

Due to the fast growth in computers and microchips, optics, lasers, solid-state detectors, artificial intelligence, and powerful software, analytical chemistry has been subjected to significant developments over the last two decades; however, still a sample preparation step is indispensable before instrumental analysis. The main reasons are the low sensitivity of the instruments, matrix interferences, and incompatibility of the sample with analytical instruments. Among the various steps of any chemical analysis, without a doubt, sample preparation is the bottleneck.^1^ On the other hand, the development of analytical chemistry is clearly moving toward the increasing application of the principles of green analytical chemistry, which requires a green protocol to be followed, especially in terms of solvent consumption during the sample preparation step.^2^ Therefore, many attempts have been made to improve the environmental friendliness of this stage, mainly through the developing approaches for miniaturized sample preparation as well as the application of new green solvents. Sample preparation techniques should be also selective, rapid, inexpensive, and simple. Among the existing sample preparation techniques, stir bar sorptive extraction (SBSE) fulfills most of the above requirements. SBSE, which was employed for the first time by the Sandra group in 2001,^3^ is an elegant enrichment technique for complex samples. A glass bar coated with an extraction medium encapsulating a magnetic stirring rod is utilized for the analyte preconcentration.^4^ A wide range of analytes were extracted by using SBSE. However, due to the requirement of selectivity of the sorbent, especially for the extraction of trace amounts of a special analyte from complex matrices, developing new SBSE coatings has become major research in SBSE development.^5^ Hasan et al. reviewed new coatings and the latest techniques for the production of SBSE coatings.^6^ Common coating materials of SBSE are poly(dimethylsiloxane), sol–gels, nanomaterials, molecularly imprinted polymers, ionic liquids, and metal–organic frameworks.^7^ Among other advantages, using viscous phases has the advantage of several orders of magnitude higher diffusion coefficients of the analytes than that of solid coatings; so, higher adsorption occurs for this type of coatings, including polymers. A stir bar with poly(dimethylsiloxane) coating is the most applied (polymeric) phase and is sold commercially under the trademark of Twister by GERSTEL GmbH & Co KG (Mülheim, Germany).^8^ However, there are no reports of using a deep eutectic solvent (DES) as a coating for SBSE.

In 2003, Abbott et al. described the invention of the first DES as a new solvent as a promising alternative to conventional organic solvents and ionic liquids.^9^ Their preparation can be simply performed by mixing two components [a hydrogen bond acceptor (HBA) and a hydrogen bond donor (HBD)] in the appropriate molar proportion. As a result of the interaction based on the formation of hydrogen bonds between the two components, the charge is relocated and the melting point of DESs is lowered in comparison to those of the pure components. DESs have unique characteristics that make them applicable in many processes, such as environmental friendliness, high conductivity, biodegradability, low flammability, low cost of components, and easy fabrication with no further purification requirement.

In 2015, a new subclass of DESs called hydrophobic DESs (hDESs) was reported by Kroon^10^ and Marrucho groups.^11^ They are immiscible with water; thus, they are capable of extracting nonpolar molecules from aqueous environments and have been suggested as potential extraction media to replace toxic organic solvents. In view of this, developing hDESs has become eminent for liquid–liquid extraction;^12^ however, to the best of our knowledge, this is the first report on using hDESs as a coating for the SBSE technique.

Vitamin D is a series of fat-soluble secosteroids that occurs in two main forms, ergocalciferol (D_2_) and cholecalciferol (D_3_). In most foodstuffs, vitamin D naturally exists as cholecalciferol. The recommended daily allowance of vitamin D for humans is 400 IU; so, accurate quantification of vitamin D in foods, supplements, and drugs is important.^13^ Because of the low concentration of vitamin D_3_ in real samples and severe interference in the complicated matrices, it is crucial to develop a protocol to be able to selectively preconcentrate and isolate it. As a result, in this research, a novel glass jacketed stir bar with an hDES coating was developed and applied for the analysis of vitamin D_3_ in various samples. The detection system was a conventional spectrophotometer. A coating was prepared by mixing tetrabutylammonium chloride and heptadecanoic acid. Microscopy images showed a uniform structure with a porous layer coating on the stir bar.

## 2 Results and Discussion

### 2.1 Optimization of Extraction

In order to achieve the highest extraction efficiency, several main factors affecting the process including extraction and desorption time, type and volume of desorption solvent, and temperature and volume of the sample were studied and optimized by three methods of one-variable-at-a-time (OVAT), central composite design (CCD) and Box–Behnken (BBD) and the results of BBD and CCD were compared. For optimization experiments, a standard solution of 230 μg L^–1^ of vitamin D_3_ was utilized.

#### 2.1.1 Effect of the Type of Eluent Solvent

Choosing a suitable elution solvent is an important parameter in the SBSE protocol. It should be able to efficiently and quickly elute vitamin D_3_ from the DES–SBSE. Solvents with proper polarity (i.e., toluene, *n*-hexane, and benzene) were examined and among them, toluene showed the highest elution efficiency and benzene showed the lowest elusion. Since toluene could elute the analyte almost completely from the SBSE coating, this solvent was considered as the eluent.

#### 2.1.2 Effect of the Volume of the Eluent

To investigate the effect of the volume of the eluent on the absorption signal, different volumes of toluene in the range of 400 μL to 1.0 mL were applied for eluting the analyte from the SB. Observations showed that using 500 μL of eluent has the best efficiency and hence this volume of the solvent was selected as the optimum volume for the next experiments.

#### 2.1.3 Effect of the Stirring Rate

The effect of the stirring rate of SB on the extraction of vitamin D_3_ was also investigated. The experimental results showed that the signal was improved with an increase in the stirring rate up to 900 rpm and then becomes constant because the maximum mass transfer of the analyte to the adsorbing phase was reached. Therefore, a stirring rate of 900 rpm was selected during the next experiments.

#### 2.1.4 Effect of Extraction and Desorption Times

The extraction efficiency of vitamin D_3_ improved by increasing the adsorption time and reached a maximum after 3 min of contacting the SB with the solution. This happens because of the saturation of the extracting phase after 3 min.

To find the optimum value for the time in which maximum vitamin D_3_ was desorbed from the hDES-coated SB, different contact times of it with 500 μL of toluene were investigated. The highest analytical signal was observed after 10 min of utilization. Increasing this time showed no improvement in the signal. As a result, 3 and 10 min were selected as the optimum extraction and desorption times, respectively.

#### 2.1.5 Effect of Sample Volume

Extraction was improved steadily by increasing the volume of sample solution from 2.5 to 5.0 mL and after then became constant. The same solution of 230 μg L^–1^ of vitamin D_3_ was utilized for this series of experiments. Due to the highest extraction efficiency, a 5.0 mL volume for the sample solution was selected for subsequent runs. In SBSE, extraction is an equilibration and not an exhaustive process; therefore, the amount of the analyte partitioning into the acceptor phase becomes independent of the sample volume when this volume is higher than the product of the partition constant and of the sorption capacity of SBSE coating.

#### 2.1.6 Effect of Temperature

The effect of temperature on both the sample and eluent on the analytical signal was studied and optimized. By changing the temperature of both the eluent and sample between 25 and 35 °C, it was demonstrated that the maximum extraction and elusion of vitamin D_3_ happens at 30 °C and then remains constant. For a 10 °C increase in the temperature, about a 20% increment in the extraction was observed. This is because an increase in eluent temperature increases the diffusion coefficient and accelerates the desorption of the analyte from the stir bar.

#### 2.1.7 Central Composite Design and Box–Behnken Design

The central composite design (CCD) and Box–Behnken design (BBD) are protocols that can be used for experimental design, investigating the effect of independent factors on the related response, achieving models, and studying techniques. In this study, significant variables selected for the extraction were adsorption time (A or X_1_), desorption time (B or X_2_), volume of the eluent (C or X_3_), and stirring rate (D or X_4_). The low, middle, and high levels of each parameter were shown as −1, 0, and +1, respectively (Tables S1 and S2). In Tables S1 and S2, the design of the actual experiments for CCD and BBD is explained. A system including four important independent factors, which predicted the absorbance by applying the quadratic equation (as a second-degree polynomial equation) for CCD and BBD, can be obtained as expressed in eq 1.
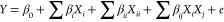
1In eq 1, *Y* is the predicted absorbance (instrument signal or dependent parameter or output), *i* and *j* are the index numbers for the pattern, β_0_ is *X*_1_, *X*_2_, *X*_3_, and *X*_4_ are coded independent parameters, β*_i_* is the linear effect, β*_ii_* is the quadratic effect, β*_ij_* proves the coefficient of the interaction factor, and ε is the random error or allows for description or uncertainties between predicted and detected data.^16,17^

A multiple regression analysis is performed to obtain the coefficients and the equation can be applied to predict the absorption.
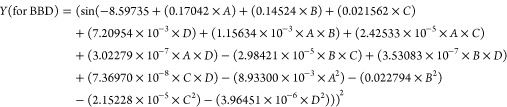
2
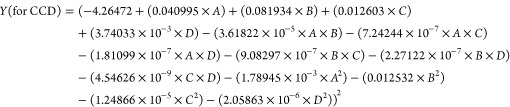
3By solving these equation systems for the condition of ∂(*Y*)/∂(*A*) = 0, ∂(*Y*)/∂(*B*) = 0, ∂(*Y*)/∂(*C*) = 0, and ∂(*Y*)/∂(*D*) = 0, the critical points in the CCD and BBD are obtained. The way of obtaining these critical points has been indicated by Santelli et al.^18^ The summary of the analysis of variance (ANOVA) is explained in Tables S3 and S4 (for BBD and CCD, respectively). The calculated data for the critical point are excesses: adsorption time (*A* or *X*_1_) = 11.3 min (for CCD) and 10.4 min (for BBD), desorption time (*B* or *X*_2_) = 3.2 min (for CCD) and 3.1 min (for BBD), volume of eluent (*C* or *X*_3_) = 504.0 μL (for CCD) and 506.0 μL (for BBD), and stirring rate (*D* or *X*_4_) = 907.0 rpm (for CCD) and 914.0 rpm (for BBD) for vitamin D_3_.

The model *F*-value of 30.92 (for BBD) and 197.10 (for CCD) showed that the models are important (proving that the quadratic model was important). A *p*-value lower than 0.001 was calculated, proving again the high importance of the regression models. There is only a 0.01% chance that a “model *F*-value” could exist because of noise. Data “Prob > F” less than 0.0500 explain model terms are important. Data greater than 0.1000 demonstrated that the model terms are not important.

The predicted *R*^2^ of 0.9810 (for CCD) and 0.8305 (for BBD) were calculated. The amount of adjusted *R*^2^ (0.9895 for CCD and 0.9352 for BBD) explained that only 1.05% (for CCD) and 6.48% (for BBD) of the total variations were not determined by the models. Good relation between the real and predicted data explained from the value of determination (*R*^2^ = 0.9946 for CCD and 0.9665 for BBD). The lack of fit determined the failure of the models to explain data in regression.

The nonsignificant value of lack of fit (>0.05) showed that the models are statistically important for the signal. The “lack of fit *F*-value” of 1.53 (for CCD) and 2.59 (for BBD) explain that the lack of fit is not important relative to the pure error.

*A*, *B*, *C*, *A*^2^, *B*^2^, *C*^2^, and *D*^2^ for CCD and *A*, *A*^2^, *B*^2^, *C*^2^, and *D*^2^ for BBD are important model terms.

**Figure 1:**
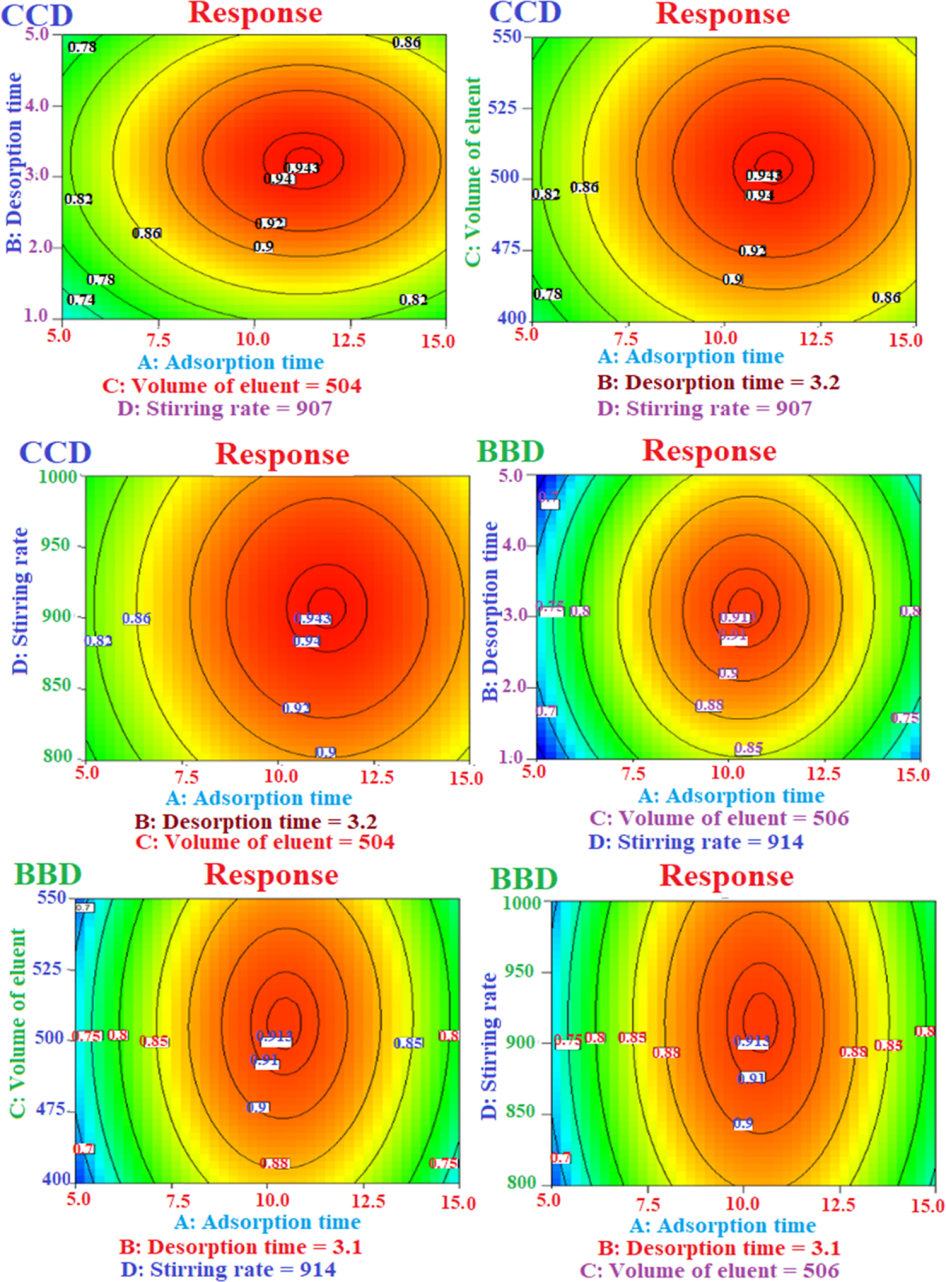
Two-dimensional (2D)/contour response explaining the effect of an independent variable on the instrument signal for vitamin D_3_.

A great degree of precision and good deal of reliability of the conducted experiments were shown by applying a small data of the coefficient of variation (CV = 2.72 for BBD and 0.80 for CCD). Figure 1 shows two-dimensional analytical response surfaces as functions of two parameters at the center level of other factors.

Equation 4 expressed that Mallow’s *C*_p_ statistic can be omitted from the response surface model. For a response surface model consisting of all terms, *C*_p_ = *p* (where *p* and *n* are the number of variables and the needed experiments in the regression model). For a response surface model using omitted terms, *C*_p_ ≈ *p* indicates a good model using little bias and *C*_p_ ≤ *p* indicates a very good prediction model. The purpose to delete terms of the response surface model to a minimum *C*_p_ ≈ *p* is achieved. If *C*_p_ > *p*, this explains that too many terms have been deleted or some remaining terms are not necessary.
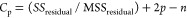
4Mallow’s *C*_p_ statistic (*C*_p_ ≈ 13.01 for CCD and 13.00 for BBD) explained a third condition (*C*_p_ ≤ *p* and *p* = 15, *n* = 30) showing a very good prediction model for CCD and BBD.

As can be seen, the results of optimization of the selected parameters by CCD and BBD are suitable and are almost similar. However, based on the predicted, adjusted, and determined *R*^2^ and CV, it can be concluded that the CCD protocol is better than that of BBD in our case. Therefore, the result of the CCD technique can be used for the following works.

### 2.2 Analytical Performance

Under the optimum extraction conditions, the analytical performance of DES–SBSE was evaluated. The linearity of the suggested technique was tested utilizing standard solutions by increasing the concentrations of vitamin D_3_. The calibration curves (absorbance vs. concentration) indicated a linear response over the 0.5–1000.0 μg L^–1^ range by equation and correlation coefficients (*R*^2^) of *A* = 3.2801C (mg L^–1^) + 0.1974 and 0.991, respectively. The limit of detection (LOD) achieved according to 3 times the standard deviation of the background for 10 experiments was 0.078 μg L^–1^. Precision explained as the relative standard deviation (RSD) for intraday repeatability and interday reproducibility of the method was better than 3.1 and 4.2%, respectively, for performing eight replicate analyses of a spiked solution standard at 50 μg L^–1^. The relative error with the mean of five replicates was 4.9% for the same amount of vitamin D_3_. A single stir bar could be used for at least 50 extractions, and the batch-to-batch reproducibility for different stir bars was <4.5%.

The obtained enrichment factor (EF), which is defined as the slope ratio of the calibration curve achieved with and without DES–SBSE, was 127-fold. In Table 1, the results obtained from the determination of vitamin D_3_ by DES–SBSE are compared with those obtained by similar approaches reported in the literature. As can be seen, despite the weakness of the low sensitivity of spectrophotometry, the DES–SBSE protocol has a suitable LOD and linear range and is comparable with sophisticated and expensive instruments such as SFC and high-performance liquid chromatography–mass spectrometry (HPLC/MS). A lower LOD is reported for method 3, which is due to the exhaustive extraction that is used in this technique. In comparison to the classical methods of extraction and some microextraction methods used for the enrichment of vitamin D_3_, DES–SBSE has lower consumption of solvents, which is in agreement with green analytical chemistry objectives. The other advantages of the developed protocol are its simplicity and lower cost of preparation of the extracting phase, which can be used many times. It also needs fewer steps during the extraction procedure in comparison to many other methods.

**Table 1:** Comparison of the Developed Method for the Determination of Vitamin D_3_ with Similar Methods

no.	extraction technique	detection method	LOD (μg L^–1^)	linear range (μg L^–1^)	recovery (%)	RSD (%)	concentration in the real sample (μg L^–1^)	ref
1	SPE on C_18_ column	SFC^a^ and reverse phase liquid chromatography/mass spectrometry	10	20–200	84.3–109.6	<7.1	Baby Ddrops (10.35–10.77 μg), vitamin AD drops (11.34–11.76 μg)	(13)
2	DLLME^b^	liquid chromatography with DAD-APMS^c^	10–500	1–100	88–103	<6.8	Spinach (ND^f^), Iceberg lettuce (ND), Cos lettuce (ND), Lamb’s lettuce (ND), infant formula (0.082 μg g^–1^), infant cereals (0.071 μg g^–1^)	(19)
3	liquid–liquid extraction	spectrophotometry	0.004	12–315	100.6–101.1	<0.14	milk (10–13), carrot (ND), egg yolk (14–16 ng g^–1^), poultry feed (ND, 0.10–0.12 ng g^–1^), mushrooms (61–65 ng g^–1^), algae (ND), pharmaceutical 200 IU (4920–4950 ng/tablet), serum (19–23 ng mL^–1^), Tuna fish (52–59 ng g^–1^)	(20)
4		HPLC-UV^d^	NM^e^	100–132,000	99.6–105.5	ND	pharmaceutical (35.04 μg g^–1^)	(21)
5	glassy carbon electrode and separation by polyethylene glycol	voltammetry	NM	0.005–1 mM	98.9–102.8	NM	0.45–0.99 mM	(22)
6	hDES–SBSE	spectrophotometry	0.08	1–1000	96.8–99.0	<4.3	soft gelatine caps (48.6), milk (without vitamin D_3_) (ND), Baby Ddrop (49.0)	this research

a SFC, supercritical fluid chromatography.

b DLLME, dispersive liquid–liquid microextraction.

c DAD-APMS, liquid chromatography with diode array and atmospheric pressure chemical ionization-mass spectrophotometry.

d HPLC-UV, high-performance liquid chromatography with an ultraviolet detector.

e NM, not mentioned.

f ND, not detected.

### 2.3 Selectivity of DES–SBSE

The common interferences that normally coexist in real samples together with vitamin D_3_ are vitamins A, K_1_, K_2_, and E, and other forms of vitamin D, including ergocalciferol (D_2_), ergosterol (provitamin D_2_), 7-dehydrocholesterol (provitamin D_3_), and calcifediol (25 (OH) D_3_). To investigate the interference of DES–SBSE for the extraction of the target analyte in samples including these interferences, aliquots of 5 mL of 230 μg L^–1^ of vitamin D_3_ were spiked by the same concentration of the interference and determined by the suggested protocol. No interferences were observed for the analysis of it, which shows the high selectivity of DES–SBSE for the analyte.

### 2.4 Analysis of the Real Sample

Since vitamin D_3_ was selected as a model analyte to evaluate the performance of this first report of DES–SBSE, some real samples containing this analyte were chosen for the determination of their vitamin D_3_ content with this method, including milk, vitamin pills, and baby Ddrops (Table 2). In order to evaluate and validate the accuracy of the suggested protocol, the samples were also spiked by vitamin D_3_ at three concentration levels of 10, 25, and 50 μg L^–1^ and the recovery was calculated. For all real spiked samples, recoveries were better than 96.8% with RSDs between 3.1 and 4.1%. Moreover, data for the analysis of the real samples were compared with a standard HPLC procedure. Student’s t-test at the 95% confidence limit approved that there is no significant difference between the two methods in terms of accuracy. Subsequently, by utilizing the DES–SBSE method, the chromatography step can be deleted.

**Table 2:** Analysis of Real Samples

sample	vitamin D_3_ added (μg L^–1^)	vitamin D_3_ found (μg L^–1^)	recovery (%)	RSD% (*n* = 3)
soft gelatine caps		48.6	97.2	1.7
	10	59.1	98.5	4.3
	25	73.8	98.4	2.7
	50	98.0	98.0	4.0
milk (without vitamin D_3_)				3.1
	10	9.7	97.0	4.1
	25	24.2	96.8	3.7
	50	48.5	97.0	3.8
Baby Ddrop		49.0	98.0	2.8
	10	59.3	98.8	2.2
	25	74.1	98.8	1.9
	50	99.0	99.0	2.5

## 3 Conclusions

In this research, a stir bar sorptive extraction coated with a hydrophobic deep eutectic solvent was fabricated and successfully applied for a highly efficient extraction of vitamin D_3_ from different matrices prior to its spectrophotometric detection. Low LOD and high precision were obtained. Using hDES largely minimized toxic organic solvent consumption and required only a few milliliters of the sample for analysis. It also has suitable linearity in a wide range of concentrations. The proposed DES–SBSE is a simple, rapid, and cost-effective protocol that does not require any preliminary derivatization step. Moreover, the hDES-coated stir bar can be fabricated straightforwardly and reused at least 50 times. No chemical modification of the surface of the SB was required. High enrichment factor and quick separation and determination (<17 min) are the other advantages of this technique. By changing the DES, it would be possible to make numerous extracting phases for different analytes. As a widely and commonly used detection method, spectrophotometric instrumentation has its own merits of simplicity, low cost, portability, and so on. To the best of our knowledge, this is the first report on using hDESs as a coating for extraction by SBSE. It has the potential that by replacing the coating with other DESs, a wide range of the other (bio)molecules selectively be extracted. A high preconcentration factor allows the analyst to determine the ultratrace amounts of such analytes in various complicated matrices.

## 4 Material and Methods

### 4.1 Chemicals and Materials

Solid pharmaceutical secondary standard of cholecalciferol (vitamin D_3_) was obtained from Sigma-Aldrich (St. Louis, MO), and a 500 mg L^–1^ stock solution of it was prepared by dissolving in toluene and stored at −20 °C. All other reagents were purchased from Merck KGaA, Darmstadt, Germany. Standard solutions were prepared daily and stored at 4 °C until the start of experiments. For the pill sample, one soft gelatine caplet (50,000 IU, containing 1250 μg of vitamin D3) was weighed (0.175 g) and dissolved in 100 mL of toluene to contain roughly 12.5 mg L^–1^ of the analyte. For DES–SBSE analysis, it was diluted to 50 μg L^–1^ with toluene. For the analysis of vitamin D_3_ in Ddrops, 125 μL of the oily sample [containing 400 IU (10 μg of vitamin D3)] was placed into a 5 mL measuring flask and diluted with hexane to mark to achieve 50 μg L^–1^ of the vitamin. Then, the procedure was performed with and without spiking. No special pretreatment was performed for the milk sample.

### 4.2 Instruments

An Uplab spectrophotometer Steroglass model Matricola 20161 (Italy) was used for the detection of vitamin D_3_ at a wavelength of 265 nm. Images of SBSE and deep eutectic solvent stir bar sorptive extraction (DES–SBSE) were taken by means of a Nikon microscope (model ECLIPSE E100) and a Nikon (model C-DS) stereomicroscope (Tokyo, Japan). A Knauer HPLC (Germany) equipped with a diode array spectrophotometer (used at a wavelength of 265 nm) and EA4300F Smartline Autosampler 3950 was employed to compare the accuracy of the method with a standard protocol. An analytical column was a Zorbax Eclipse ODS non-end-capped (25 cm × 0.46 cm × 5 μm) (Agilent). ChromGate V3.1.7 software was utilized for chromatographic data handling. The flow rate was 1.0 mL min^–1^ with an injection loop volume of 20 μL. The mobile phase was a gradient consisting of water, acetonitrile, and isopropanol starting with acetonitrile:water 70:30 v/v and ending with 60:40 v/v acetonitrile:isopropanol in 14 min.

### 4.3 Preparation of SBSE–DESs

**Figure 2:**
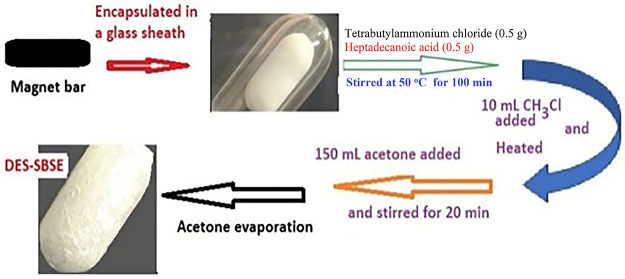
Preparation of DES–SBSE.

**Figure 3:**
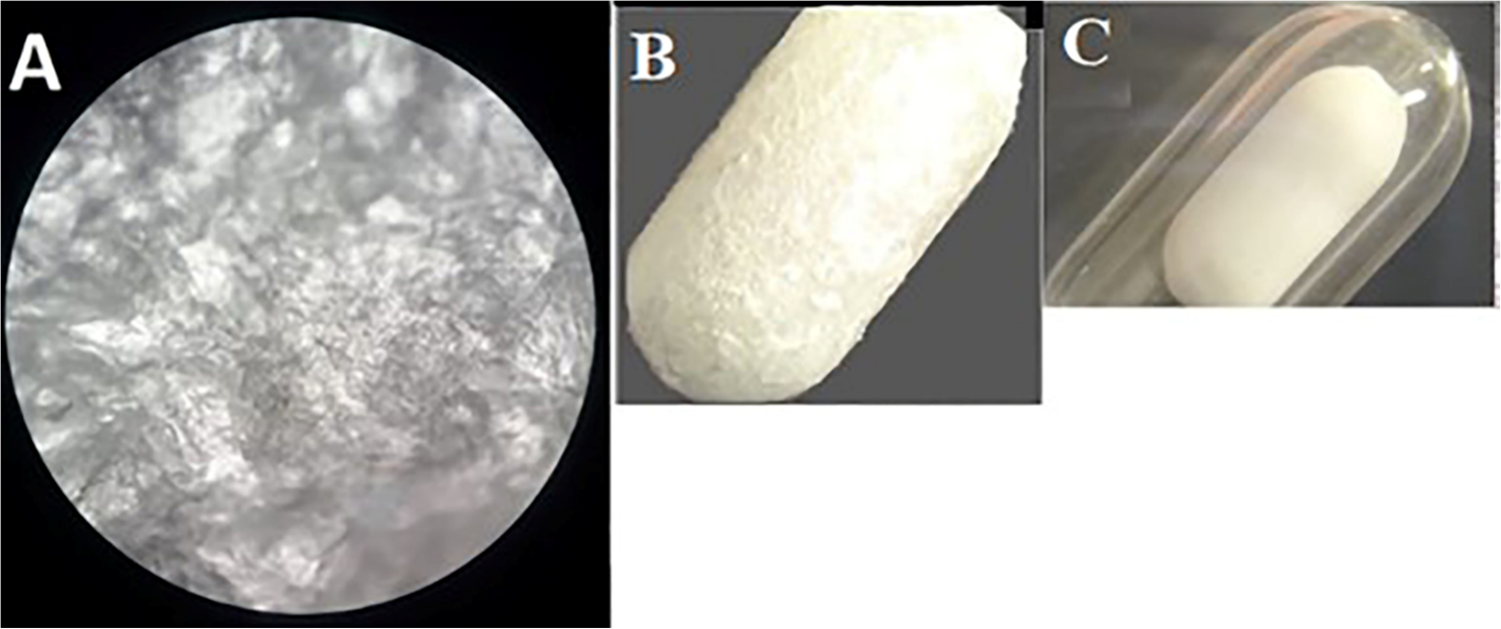
Images of prepared DES–SBSE. Coated (A, B) and without coating (c). Image (a) is taken with an optical microscope (40× magnification) and images B and C are taken by a stereomicroscope (4× magnification).

In 2020, Momotko et al. suggested a method for the preparation of a DES-based stationary phase for gas chromatography.^14^ Here, the same procedure was followed but customized for SBSE. A magnetic iron bar (1.0 cm × 2 mm) was placed inside a hollow glass bar and was sealed by flame to make the stir bar. A 0.50 g of each tetrabutylammonium chloride (HBA) and heptadecanoic acid (HBD) were weighed and placed in a water bath at 50 °C and a stir bar was placed in it. The vial was sealed and stirred for 100 min at 900 rpm until a homogeneous liquid phase was achieved. Then, 10 mL of CH_3_Cl was added and the mixture was heated up to near boiling while stirring. After that, 150 mL of acetone was added to the mixture and stirred further for 20 min. Finally, it was left at room temperature until the solvent evaporated (Figure 2). Figure 3 shows the real images of the glass stir bar before and after coating with hDES. As can be seen, a porous layer of DES is uniformly coated on it with a thickness of almost 1.3 mm. The film thickness was measured by using a caliper by differentiation of the thickness of DES–SBSE and the glass stir bar thickness based on the method described by Amlashi and Hadjmohammadi.^15^

### 4.4 Extraction Procedure

**Figure 4:**
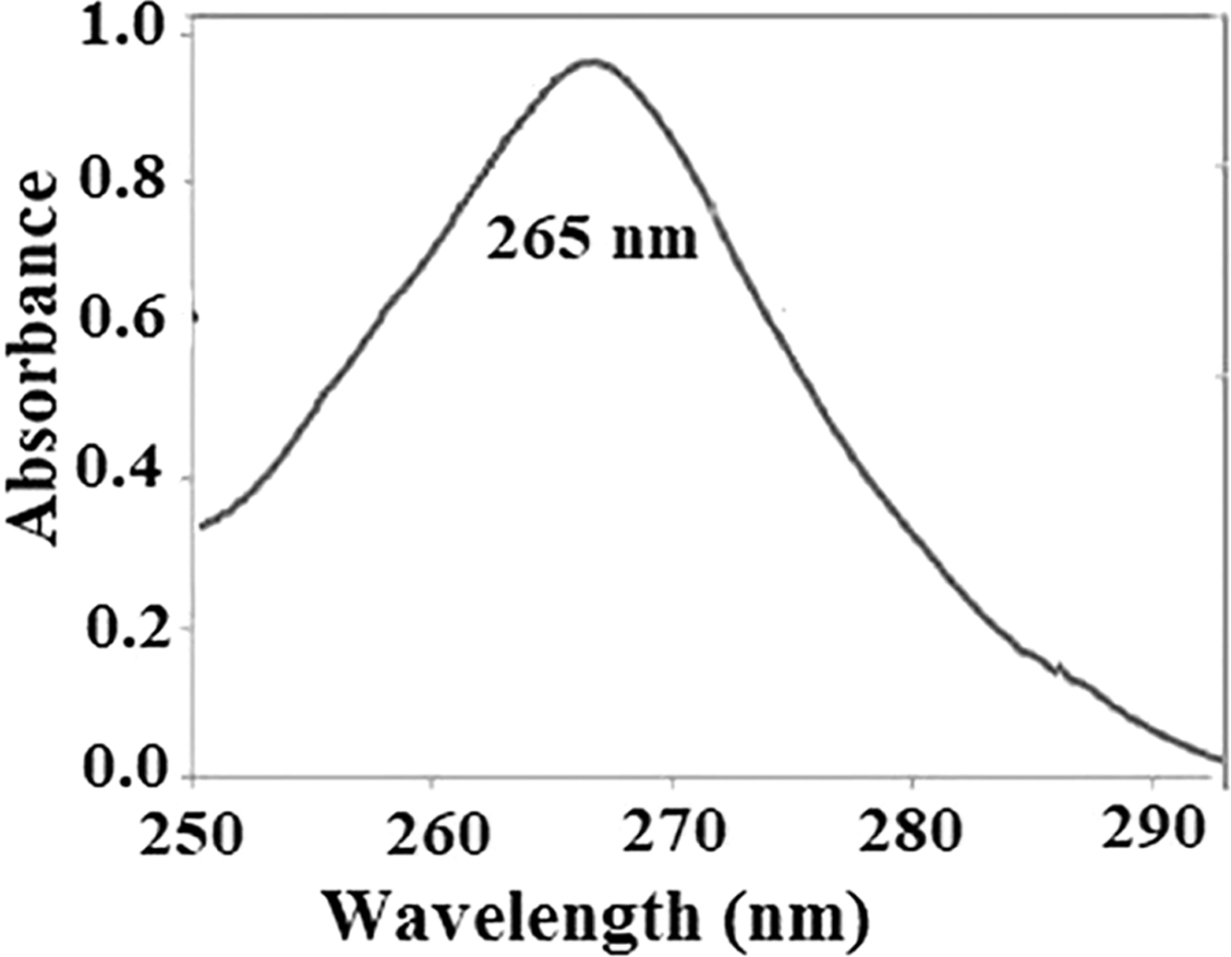
Absorbance spectra of 230 μg L^–1^ of vitamin D_3_ against blank regent after DES–SBSE (extraction conditions: sample solution 5.0 mL, stirring rate 900 rpm, 10 min of extraction, desorption time 3 min, temperature 30 °C).

SBSE was performed in two convenient steps. For the adsorption of the analyte, the hDES-coater stir bar was placed into 5.0 mL of the sample solution and the mixture was stirred for 10.0 min at 900 rpm. In this step, vitamin D_3_ is extracted from the hDES. For desorption of the analyte, SB was immersed for 3.0 min into a vial containing 500 μL of toluene (30 °C) as an eluent solvent. DES–SB was removed and the toluene phase was transferred to a spectrophotometer cuvette. To ensure no memory effect, DES–SBSE was washed several times with 1 mL of toluene before the next use. Figure 4 shows the absorbance spectra of 230 μg L^–1^ of vitamin D_3_ against blank regent after DES–SBSE.

## Supporting Information Available

Results of central composite design and Box–Behnken design methods for optimization of vitamin D_3_ by deep eutectic solvent stir bar sorptive extraction (PDF)
