# Supplementary material for: Deep Eutectic Solvent Stir Bar Sorptive Extraction: A Rapid Microextraction Technique for the Determination of Vitamin D3 by Spectrophotometry
Source: ACS Omega. 2023 Jun 23;8(26):23643–50. doi: 10.1021/acsomega.3c01670 (PMC10324053; doi:10.1021/acsomega.3c01670)
Supplement: Supplementary file 1 — ao3c01670_si_001.pdf [file ao3c01670_si_001.pdf]

Supplementary data to:

Deep eutectic solvent stir bar sorptive extraction: a rapid microextraction technique for determination of vitamin D_3 by spectrophotometry

*Sayyed Hossein Hashemi,[a] Massoud Kaykhaii,[b,] * Ahmad Jamali Keikha[c] and Leila Raisi [a]*

[a]Department of Marine Chemistry, Faculty of Marine Science, Chabahar Maritime University, Chabahar, Iran.

[b]Department of Process Engineering and Chemical Technology, Faculty of Chemistry, Gdańsk University of Technology, Narutowicza 11/12, Gdansk 80–233, Poland.

Tel: +48731960312; Fax: +48583472065; E-mail: massoud.kaykhaii@pg.edu.pl

[c]Department of Mechanical Engineering, Faculty of Marine Engineering, Chabahar Maritime University, Chabahar, Iran.

Table S1. Central composite design (CCD) of independent variables and their corresponding runs and predicted data

Adsorption Time	Desorption Time	Volume of Eluent	Stirring Rate	Real response	Predicted response	Error%
5.0	1.0	450	800	0.60	0.60	0.00
5.0	1.0	450	1000	0.61	0.61	0.00
5.0	1.0	550	800	0.62	0.62	0.00
5.0	1.0	550	1000	0.63	0.63	0.00
5.0	3.0	500	900	0.83	0.81	2.41
5.0	5.0	450	800	0.64	0.64	0.00
5.0	5.0	450	1000	0.65	0.65	0.00
5.0	5.0	550	800	0.66	0.66	0.00
5.0	5.0	550	1000	0.67	0.67	0.00
10.0	1.0	500	900	0.83	0.82	1.20
10.0	3.0	450	900	0.88	0.87	1.14
10.0	3.0	500	800	0.89	0.89	0.00
10.0	3.0	500	900	0.95	0.94	1.05
10.0	3.0	500	900	0.94	0.94	0.00
10.0	3.0	500	900	0.93	0.94	-1.08
10.0	3.0	500	900	0.92	0.94	-2.17
10.0	3.0	500	900	0.95	0.94	1.05
10.0	3.0	500	900	0.94	0.94	0.00
10.0	3.0	500	1000	0.90	0.90	0.00
10.0	3.0	550	900	0.87	0.89	-2.30
10.0	5.0	500	900	0.85	0.86	-1.18
15.0	1.0	450	800	0.68	0.68	0.00
15.0	1.0	450	1000	0.69	0.69	0.00
15.0	1.0	550	800	0.70	0.70	0.00
15.0	1.0	550	1000	0.71	0.71	0.00
15.0	3.0	500	900	0.87	0.89	-2.30
15.0	5.0	450	800	0.72	0.72	0.00
15.0	5.0	450	1000	0.73	0.73	0.00
15.0	5.0	550	800	0.74	0.73	1.35
15.0	5.0	550	1000	0.75	0.74	1.33
7	2	460	850	0.83	0.79	4.82
8	3	480	900	0.85	0.89	-4.71
9	4	500	950	0.87	0.90	-3.45
10	5	510	1000	0.80	0.83	-3.75
12	2	520	860	0.86	0.89	-3.49

14	3	560	880	0.81	0.84	-3.70
16	3	500	910	0.90	0.87	3.33
4	3	500	920	0.79	0.77	2.53
10	6	490	960	0.77	0.75	2.60
10	3	470	1050	0.81	0.83	-2.47

Table S2. Box- Behnken design (BBD) of independent variables and their corresponding runs and predicted data

Adsorption Time	Desorption Time	Volume of Eluent	Stirring Rate	Real Response	Predicted response	Error%
5.0	1.0	500	900	0.64	0.63	1.56
5.0	3.0	450	900	0.65	0.66	-1.54
5.0	3.0	500	800	0.66	0.67	-1.52
5.0	3.0	500	1000	0.67	0.69	-2.99
5.0	3.0	550	900	0.68	0.67	1.47
5.0	5.0	500	900	0.65	0.63	3.08
10.0	1.0	450	900	0.78	0.79	-1.28
10.0	1.0	500	800	0.81	0.81	0.00
10.0	1.0	500	1000	0.82	0.83	-1.22
10.0	1.0	550	900	0.80	0.82	-2.50
10.0	3.0	450	800	0.85	0.83	2.35
10.0	3.0	450	1000	0.88	0.85	3.41
10.0	3.0	500	900	0.90	0.91	-1.11
10.0	3.0	500	900	0.91	0.91	0.00
10.0	3.0	500	900	0.90	0.91	-1.11
10.0	3.0	500	900	0.93	0.91	2.15
10.0	3.0	500	900	0.91	0.91	0.00
10.0	3.0	500	900	0.92	0.91	1.09
10.0	3.0	550	800	0.86	0.85	1.16
10.0	3.0	550	1000	0.89	0.87	2.25
10.0	5.0	450	900	0.80	0.81	-1.25
10.0	5.0	500	800	0.82	0.82	0.00
10.0	5.0	500	1000	0.83	0.84	-1.20
10.0	5.0	550	900	0.81	0.83	-2.47
15.0	1.0	500	900	0.71	0.68	4.23
15.0	3.0	450	900	0.70	0.72	-2.86
15.0	3.0	500	800	0.72	0.74	-2.78
15.0	3.0	500	1000	0.73	0.76	-4.11
15.0	3.0	550	900	0.75	0.75	0.00

15.0	5.0	500	900	0.76	0.72	5.26
7	2	460	850	0.74	0.78	-5.40
8	3	480	900	0.86	0.87	-1.16
9	4	500	950	0.92	0.89	3.26
10	5	510	1000	0.89	0.84	5.62
12	2	520	860	0.91	0.87	4.40
14	3	560	880	0.74	0.79	-6.76
16	3	500	910	0.67	0.70	-4.48
4	3	500	920	0.59	0.62	-5.08
10	6	490	960	0.80	0.77	3.75
10	3	470	1050	0.87	0.85	2.30

Table S3. ANOVA analysis for response vitamin D_3 (CCD)

Source	Sum of Squares	df	Mean Square	F Value	p-value Prob > F	% PC= (SS/ ∑ SS) × 100
Model	0.136086	14	0.00972	197.0992	< 0.0001	
A-Adsorption Time	0.009409	1	0.009409	190.7758	< 0.0001	33.94545
B-Desorption Time	0.002357	1	0.002357	47.78331	< 0.0001	8.5035
C-volume of Eluent	0.000472	1	0.000472	9.574763	0.0074	1.702865
D-Stirring Rate	0.000162	1	0.000162	3.291735	0.0897	0.584458
AB	2.09×10^{-6}	1	2.09×10^{-6}	0.042473	0.8395	2.09×10^{-6}
AC	5.25×10^{-7}	1	5.25×10^{-7}	0.010636	0.9192	5.25×10^{-7}
AD	1.31×10^{-7}	1	1.31×10^{-7}	0.00266	0.9595	1.31×10^{-7}
BC	1.32×10^{-7}	1	1.32×10^{-7}	0.002677	0.9594	1.32×10^{-7}
BD	3.3×10^{-8}	1	3.3×10^{-8}	0.000669	0.9797	3.30×10^{-8}
CD	8.27×10^{-9}	1	8.27×10^{-9}	0.000168	0.9898	8.27×10^{-9}
A^2	0.005185	1	0.005185	105.1412	< 0.0001	18.70626
B^2	0.00651	1	0.00651	132.0121	< 0.0001	23.48654
C^2	0.002525	1	0.002525	51.19396	< 0.0001	9.109604
D^2	0.001098	1	0.001098	22.2642	0.0003	3.961325
Residual	0.00074	15	4.93×10^{-5}			
Lack of Fit	0.000557	10	5.57×10^{-5}	1.525873	0.3351	not significant
Pure Error	0.000183	5	3.65×10^{-5}			
Cor Total	0.136826	29				

PC%: Percent contribution, SS: Sum of squares

Table S4. ANOVA analysis for response vitamin D_3 (BBD)

Source	Sum of Squares	df	Mean Square	F Value	p-value Prob > F	% PC= (SS/ ∑ SS) × 100
Model	0.391234	14	0.027945	30.91693	< 0.0001	
A-Adsorption Time	0.017439	1	0.017439	19.29325	0.0005	3.851931
B-Desorption Time	0.001416	1	0.001416	1.56694	0.2298	0.312766
C-volume of Eluent	0.001999	1	0.001999	2.211719	0.1577	0.44154
D-Stirring Rate	0.001569	1	0.001569	1.735956	0.2074	0.346561
AB	0.000535	1	0.000535	0.591722	0.4537	0.118171
AC	0.000147	1	0.000147	0.162694	0.6924	0.032469
AD	9.1414×10^{-8}	1	9.14×10^{-8}	0.000101	0.9921	9.1414×10^{-8}
BC	3.56×10^{-5}	1	3.56×10^{-5}	0.03941	0.8453	3.56×10^{-5}
BD	1.99×10^{-8}	1	1.99×10^{-8}	2.21×10^{-5}	0.9963	1.99×10^{-8}
CD	5.43×10^{-7}	1	5.43×10^{-7}	0.000601	0.9808	5.43×10^{-7}
A^2	0.341993	1	0.341993	378.3605	< 0.0001	75.5395
B^2	0.057005	1	0.057005	63.06654	< 0.0001	12.59128
C^2	0.019853	1	0.019853	21.96394	0.0003	4.385136
D^2	0.010778	1	0.010778	11.9237	0.0035	2.380647
Residual	0.013558	15	0.000904			
Lack of Fit	0.011363	10	0.001136	2.587502	0.1528	not significant
Pure Error	0.002196	5	0.000439			
Cor Total	0.404792	29				

% PC=Percent contribution, SS:sum of squares
